# Public Safety Heroes (PUSH) Workout: Task-Specific High-Intensity Functional Training for Emergency Readiness in Fire and Police—Proof of Concept

**DOI:** 10.3390/jfmk11010060

**Published:** 2026-01-30

**Authors:** Roberto Barcala-Furelos, Fernando Zarzosa-Alonso, Martín Otero-Agra, Felipe Fernández-Méndez, Alejandra Alonso-Calvete

**Affiliations:** 1 Faculty of Education and Sports Sciences, University of Vigo, 36005 Pontevedra, Spain; fzarzosa@uvigo.es; 2 REMOSS Research Group, University of Vigo, 36005 Pontevedra, Spain; martinoteroagra@gmail.com (M.O.-A.); felipefernandez@uvigo.gal (F.F.-M.); alejalonso@uvigo.es (A.A.-C.); 3 School of Nursing of Pontevedra, University of Vigo, 36005 Pontevedra, Spain; 4 Faculty of Physiotherapy, University of Vigo, 36005 Pontevedra, Spain

**Keywords:** high-intensity functional training, chest compressions, cardiopulmonary resuscitation, public safety, metabolic stress, emergency readiness

## Abstract

**Objectives**: Public safety personnel, such as police and firefighters, face high physical demands during emergencies, including compressions-only cardiopulmonary resuscitation (CPR) under fatigue. This study aimed to evaluate a High-Intensity Functional Training (HIFT) program, the Public Safety Heroes Workout (PUSH), designed to enhance emergency readiness. **Methods**: Fifteen active-duty public safety officers participated in a pilot study. The PUSH workout included tasks like sandbag carries and burpee box jumps, interspersed with chest compressions (CC). Physiological responses, including lactate levels, heart rate, and Compressions-only CPR quality, were measured. **Results**: The PUSH workout induced significant metabolic stress, with lactate levels rising from 2.2 to 14.8 mmol·L^−1^. Heart rates peaked at 170 bpm, indicating high cardiovascular demand. Compressions-only CPR quality remained high, with firefighters outperforming police in compression rate. **Conclusions**: The PUSH workout effectively simulates the physical demands of emergency situations, enhancing readiness and compressions-only CPR performance under fatigue. This training model could be applied to other first responders and CrossFit^®^ athletes.

## 1. Introduction

Public safety personnel—including law enforcement officers (police), firefighters, and other first responders—routinely confront situations that demand immediate action to preserve life. On a daily basis, they must be prepared to manage life-threatening emergencies [1]. In many instances, they are the first to respond to out-of-hospital cardiac arrest, providing critical care until specialized teams arrive [2,3]. Early initiation of Basic Life Support (BLS) by public safety personnel has been associated with improved survival, particularly survival with favorable neurological outcomes [3].

The occupational demands placed on fire and police personnel differ markedly from those of other first responders or Emergency Medical Services (EMS). Their roles often require high levels of physical exertion, including running, navigating obstacles, climbing, and dragging loads [4]. These demands become especially salient in rescue scenarios that necessitate substantial physical effort and in the delivery of cardiopulmonary resuscitation (CPR) under conditions of prior fatigue—an important consideration [5], as fatigue is, at times, a limiting factor during CPR [6], especially in the compressions-only CPR modality [7].

Physical fitness is a set of attributes that people possess or attain, defined as the ability to perform daily tasks with vigor and alertness [8]. Physical fitness may therefore play a critical role in the timeliness and effectiveness of the initial response to disasters and emergencies [9]. Among the key challenges in this domain is the need for training paradigms specifically adapted to working with heavy loads and complex, task-specific movements [10]. The integration of physical fitness and task-specific operational readiness makes sense in professions linked to first responders (such as firefighters, police officers, or lifeguards) where it is not uncommon to perform rescues or first aid maneuvers like CPR under conditions of physical fatigue and high physiological demands [4,5,11].

Ensuring adherence to such training requires not only adequate motivation but also a pragmatic balance between available time and workout duration. In contemporary fitness practice, High-Intensity Functional Training (HIFT) has gained notable prominence, with CrossFit^®^ (CrossFit, Inc., Washington, DC, USA) emerging as a globally successful model [12]. CrossFit^®^-based approaches are known to promote a range of physiological adaptations, including metabolic, cardiovascular, and muscular strength improvements [13,14,15], and they typically require athletes to sustain high intensity, particularly when performing the greatest possible number of repetitions within a fixed time—an approach commonly termed “as many repetitions as possible” (AMRAP).

Against this background, we conducted an exploratory, proof-of-concept assessment to examine whether a HIFT-style workout—tailored to the emergency tasks of fire and police personnel and structured as a ‘workout of the day’ (WOD)—could provide a time-efficient training methodology with substantial physiological impact.

Accordingly, the aim of this study was to design and evaluate a HIFT program to explore acute physiological responses and to incorporate the elements most representative of public safety tasks (including CPR) performed by first responders in out-of-hospital emergencies.

## 2. Materials and Methods

### 2.1. Participants

Fifteen public safety officers (one group of 8 law enforcement personnel—police and military personnel—and a second group of 7 firefighters) participated voluntarily in this research. As an inclusion criterion, all participants were active-duty and engaged in structured fitness programs (CrossFit^®^ athletes for at least the past two years, with a frequency of at least three training sessions per week). None reported injuries or cardiovascular contraindications. This profile reflects a highly trained subgroup of public safety personnel and should be interpreted as applicable to well-conditioned officers.

Their demographic characteristics were Mean ± SD age: 34.1 ± 5.2 years; height: 177.3 ± 6.5 cm; body mass: 80.6 ± 8.7 kg. Written consent was obtained in accordance with the Declaration of Helsinki. The protocol was approved by the institutional ethics committee of the Faculty of Education and Sport Sciences at the University of Vigo, code 03-151025.

### 2.2. Training Design

**Figure 1:**
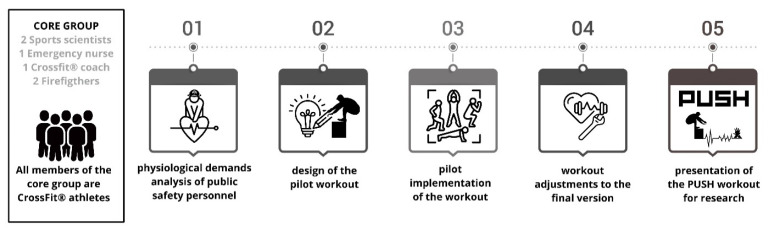
Workout design process.

A core group of six experts participated in the design (two sports scientists, one emergency nurse, two firefighters, and one CrossFit^®^ coach). All members of this team had experience in high-intensity training based on CrossFit^®^ and in training first responders. Through a focus group, a workout was designed—called in CrossFit^®^ terminology ‘workout of the day’ (WOD)—that integrated the main physical activities that can be performed during a rescue or an emergency: lifting, carrying, and dropping a victim; jumping an obstacle; walking/running; standing up/down (as in a burpee); and CPR in its chest-compressions-only version (CC), known as compressions-only CPR. The creation and development of this workout consisted of five phases, which can be seen in Figure 1.

**Figure 2:**
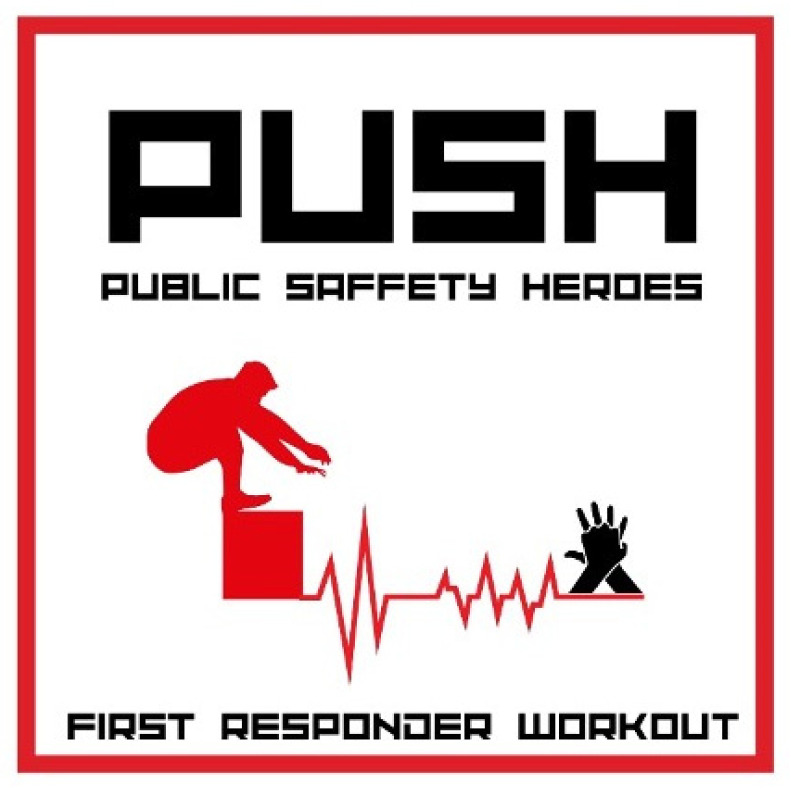
Public Safety Heroes Workout (PUSH) logo.

Before administering the WOD in this study, the researchers and other collaborators who were not part of the sample tested the workout, making the necessary adjustments to tasks, repetitions, and timing to create a WOD tailored to the high-intensity demands of emergencies faced by public safety personnel as first responders. The final proposal was called “Public Safety Heroes Workout,” and the acronym PUSH was created to refer to this WOD (Figure 2).

This workout simulates operational load carriage and obstacle negotiation (Figure 2). A metabolic conditioning (METCON) in this WOD, the participants lifted a 60 kg sandbag from the ground, carried it 10 m, dropped it, turned, and repeated the task for five carries (50 m total). They then performed 10 burpee box jumps over a 1.20 m obstacle (two stacked 60 cm boxes). Subsequent rounds involved four carries (40 m) and eight burpee box jumps, followed by three carries (30 m) and six burpee box jumps.

**Figure 3:**
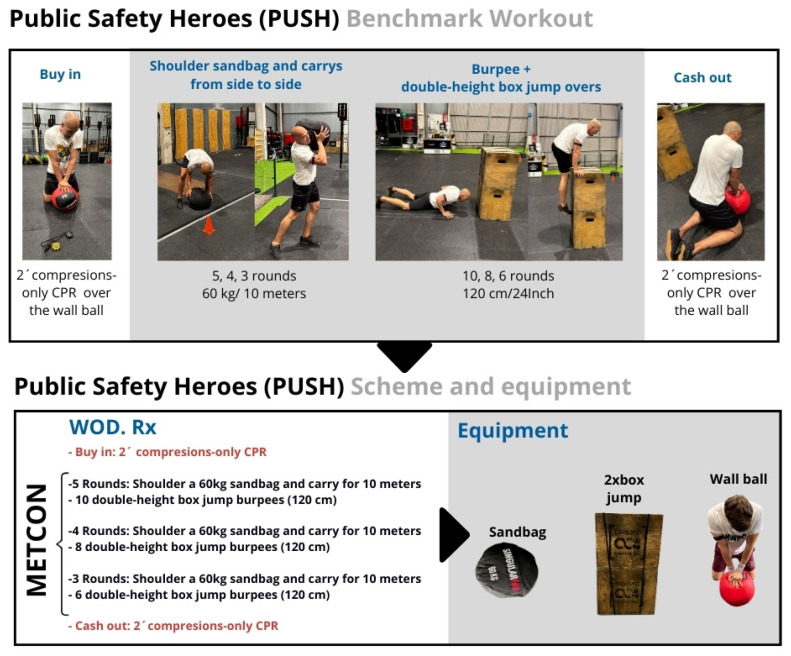
Public Safety Heroes Workout (PUSH) design.

Participants should perform an initial 2 min compressions-only CPR test (called “buy-in” in CrossFit^®^ terminology), followed by the WOD, and then a final 2 min compressions-only CPR (called “cash-out” in CrossFit^®^ terminology) (Figure 3).

The total effort for this WOD (with compressions-only CPR) required approximately 10 min, depending on individual pacing.

### 2.3. Experimental Design

A within-subject pre–post pilot study was conducted to evaluate the acute physiological demands and compression-only CPR performance associated with a functional workout (PUSH) designed for the physical training of public safety personnel and first responders. As a proof-of-concept study, the primary aim was not hypothesis testing but to describe feasibility and acute physiological responses to this task-specific high-intensity functional training protocol.

The sessions were conducted on separate days and organized by occupational group: Group 1, law enforcement personnel (Spanish National Police and Civil Guard), and Group 2, firefighters. To ensure standardized testing conditions, all trials were carried out at Box 004 Fitness Center in Pontevedra, Spain. Environmental conditions were maintained at 20–24 °C and 60–70% relative humidity throughout all sessions.

### 2.4. Procedures and Variables

#### 2.4.1. Procedures

Each participant performed two compressions-only CPR tests during 2 min: one before and one immediately after completing the METCON. The compressions-only CPR trials were conducted on a 6 kg medicine ball (wall ball), with an electronic feedback device placed on top of the ball to record compression depth, rate, and full recoil. To replicate real emergency conditions, the WOD was performed without a prior warm-up, reflecting the sudden physical demands that first responders face during actual interventions.

#### 2.4.2. Variables

- Routine time (WOD for time): Two measures were collected in this section: (a) total WOD time (including compressions-only CPR) and (b) METCON time. This variable was recorded in seconds (s) using an arm sensor (Polar Verity Sense®, Polar Electro Oy, Kempele, Finland).
- Lactate: Capillary blood samples were collected from the fingertip using a portable analyzer (LactateScout, SensLab GmbH, Leipzig, Germany), with results expressed in mmol·L^−1^, immediately before the first 2 min compressions-only CPR bout and 1 min after the second 2 min compressions-only CPR bout.
- Rating of Perceived Exertion (RPE): RPE was recorded at five time points using the modified Borg 0–10 scale: (1) before starting the session, (2) immediately after the first CPR bout, (3) after completing the WOD, (4) after the second CPR bout, and (5) during the final lactate sampling (3 min post-WOD). This enabled tracking of perceived effort throughout the protocol.
- Heart rate (HR): HR was continuously recorded using an optical arm sensor (Polar Verity Sense®, Polar Electro Oy, Kempele, Finland). Mean and peak HR values were extracted for analysis.
- Compressions-only CPR quality in percentage (%): CC were performed on a 6 kg medicine ball, with a Laerdal CPRmeter (Stavanger, Norway) placed on top to record compression depth, rate, and full release in real time. The same wall ball and setup were used for all participants to ensure standardization. Athletes received real-time feedback on their CC to standardize effort, aiming to maintain the European Resuscitation Council Guidelines for Resuscitation, Adult Basic Life Support 2025 (ERCGR2025) [16] recommendations for CC: a compression rate of 100–120 per minute, a depth of 5–6 cm, and complete chest recoil.

**Figure 4:**
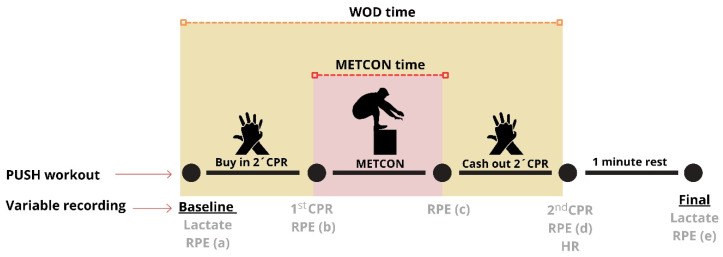
Timing of each variable’s recording during the workout.

The timing of each variable’s recording can be seen in Figure 4.

### 2.5. Statistical Analysis

All analyses were conducted using IBM SPSS Statistics, version 21 for Windows (Armonk, NY, USA). Results are reported as measures of central tendency (median) and dispersion (interquartile range, IQR). For between-group comparisons (across different types of first responders), after assessing normality with the Shapiro–Wilk test, we used either the independent samples *t* test (parametric) or the Mann–Whitney U test for independent samples (nonparametric), as appropriate. The variables that met the assumptions of normality, for which parametric tests were used, were Baseline lactate, Post-WOD lactate, Baseline RPE, Post BUY-IN RPE, Mean HR, Maximal HR, BUY-IN CC with adequate rate, CASH-OUT CC with adequate rate, and CASH-OUT Quality of CC. Nonparametric tests were used for the remaining variables. For within-group comparisons (i.e., comparing different time points for the same participants, either in the full sample or within each first responder group), after assessing normality with the Shapiro–Wilk test, we used either the paired samples *t* test (parametric) or the Wilcoxon signed-rank test for paired samples (nonparametric), as appropriate in the case of the RPE variable, in which multiple paired comparisons were made, and the Friedman repeated-measures test (nonparametric) with Bonferroni correction was used. A significance level of *p* < 0.05 was set for all analyses.

## 3. Results

### 3.1. Routine Time (WOD for Time)

The median time to complete the WOD was 607 [585–675] s. Police took 625 [578–675] s versus firefighters who took 605 [590–690] s, *p* = 1.0. The METCON execution time was 363 [328–416] s. Police reached 359 [322–416] s, versus firefighters at 363 [343–420] s, *p* = 0.82.

### 3.2. Physiological Variables (Table 1)

#### 3.2.1. Lactate Measurements (mmol·L^−1^)

Lactate increased significantly (*p* < 0.001) from 2.2 [1.9–3.1] mmol·L^−1^ at baseline to 14.8 [13.8–15.4] mmol·L^−1^ post-workout, confirming a high glycolytic demand. No significant differences were observed between occupational groups (*p* > 0.05).

**Table 1:** Physiological variables: Lactate, RPE and HR.

Variables		Public Safety (*n* = 15) Median (IQR)	Police (*n* = 8) Median (IQR)	Fire (*n* = 7) Median (IQR)	*p*-Value (ES)
Lactate (mmol·L^−1^)	Baseline lactate	2.2 (1.9–3.1)	1.95 (1.2–2.5)	3.0 (2.4–3.3)	0.06 (1.05) *
	Post-WOD lactate	14.8 (13.8–15.4)	14.2 (13.1–14.6)	15.0 (14.9–17.1)	0.16 (0.78) *
	Lactate comparison	<0.001 (4.34) †	<0.001 (4.16) †	<0.001 (4.48) †	
	Baseline (a)	2 (1–3)	2 (1–3)	2 (2–3)	0.61 (0.27) *
RPE (0–10 scale)	Post-BUY-IN (b)	4 (3–6)	4 (3–6)	5 (3–6)	0.65 (0.24) *
	Post-WOD (c)	8 (8–9)	8 (8–9)	8 (8–9)	0.79 (0.07) ^ỻ^
	Post-CASH-OUT (d)	7 (7–8)	8 (7–9)	7 (7–8)	0.71 (0.10) ^ỻ^
	3 min post-WOD (e)	5 (5–6)	5 (5–7)	5 (5–6)	0.61 (0.13) ^ỻ^
	RPE comparison	a vs. c < 0.001 (1.71) ǂ a vs. d < 0.001 (1.42) ǂ a vs. e = 0.022 (0.79) ǂ b vs. c = 0.008 (0.86) ǂ b vs. d < 0.001 (1.16) ǂ c vs. e = 0.003 (0.92) ǂ	a vs. c < 0.001 (1.68) ǂ a vs. d < 0.001 (1.45) ǂ b vs. c = 0.012 (1.15) ǂ	a vs. c < 0.001 (1.76) ǂ a vs. d = 0.003 (1.37) ǂ b vs. c = 0.018 (1.18) ǂ	
	Mean HR	152 (145–159)	152 (140–161)	157 (150–159)	0.80 (0.13) *
HR	Maximal HR	170 (163–175)	172 (163–179)	170 (161–172)	0.51 (0.35) *

RPE: Rate of Perceived Exertion. HR: Heart rate. IQR: Interquartile Range. ES: Effect size. * Independent samples *t* test (in brackets, effect size calculated with Cohen’s d), ^ỻ^ Mann–Whitney U (in brackets, effect size calculated with Rosenthal’s r), † paired samples *t* test (in brackets, effect size calculated with Paired Cohen’s d), ǂ Friedman test for related samples with Bonferroni correction (in brackets, effect size calculated with Rosenthal’s r). Significance level of *p* < 0.05 for all analysis.

#### 3.2.2. Rating of Perceived Exertion (RPE; 0–10 Scale)

RPE was recorded at five time points. Baseline RPE was 2 [1–3] and increased to 4 [3–6] immediately after the first compressions-only CPR (buy-in). Compared with baseline, RPE rose significantly at the end of the METCON, with public safety personnel reaching 8 [8–9] (*p* < 0.001), and remained elevated after the second compressions-only CPR at the end of the WOD, at 7 [7–8] (*p* < 0.001). Three minutes’ post-workout, RPE decreased to 5 [5–6] (*p* = 0.022). Across occupational groups, a common pattern was observed: a significant increase from baseline to the end of the METCON (highest recorded value) and to the end of the WOD.

#### 3.2.3. Heart Rate (Beats per Minute, bpm)

The median of mean HR during the workout was 152 [145–159] bpm, while peak HR reached approximately 170 [163–176] bpm, corresponding to about 90–95% of maximal HR (HRmax). No significant differences were found between occupational groups (*p* > 0.05).

### 3.3. Chest Compression Variables

This study evaluated the effectiveness of CC in a baseline situation (Buy-In) at the start of the workout and at the end of the workout (Cash-Out). The outcomes were measured in terms of adequate compression depth, release, rate, and overall quality, with means and confidence intervals calculated for each. The overall compressions-only CPR score for the entire public safety sample for first test achieved a median of 93 [90–95] %, while in a second had a median of 94 [90–96] %, with a *p* = 0.10. In the comparison between police and firefighters, no significant differences were found in the intragroup comparison. However, significant differences were found in the intergroup comparison, where firefighters achieved superior quality compared to police, both at the start of the workout (compressions-only CPR in Buy-in, *p* = 0.011) and at the end of the workout (compressions-only CPR in Cash-out, *p* = 0.016). In the other compression variables (depth, release, and rate), statistically significant differences were only found in the rate. Firefighters maintained a higher rate of correct compressions compared to police in both compressions-only CPR test: Buy-in (+6%, *p* = 0.02) and Cash-out (+10%, *p* = 0.002) (Table 2).

The graphical representation of the results can be found in the online Supplementary Figure S1.

## 4. Discussion

The objective of this study was to design and evaluate a HIFT program, as a proof of concept, that imposed a substantial metabolic demand and incorporated elements representative of public safety tasks performed by first responders during out-of-hospital emergencies.

The main findings were as follows:

The PUSH protocol elicited a high physiological response in terms of blood lactate concentration, perceived fatigue, and HRmax peaks, making it a workout consistent with the intensity demands of rescue, assistance, and public safety interventions. Based on this descriptive acute study, public safety personnel with prior experience and a solid baseline of physical conditioning demonstrated the ability to maintain high-quality CC with feedback, even under conditions of fatigue and submaximal lactacidemia. These findings describe observed acute performance and do not imply training effects or causal adaptations.

In the scientific literature on training for public officers, numerous fitness tests have been described. Many target specific components such as strength, aerobic capacity, or muscular endurance. Some use decontextualized tasks (e.g., weightlifting), whereas others use more realistic conditions [17]. To our knowledge, this was the first investigation to analyze a HIFT program based on a methodology similar to CrossFit^®^ that integrates tasks such as CPR.

Why integrate everything within a training model based on CrossFit^®^ methodology? CrossFit^®^ is one of the fastest-growing training models [12]. It incorporates multiple physical domains within a unified structure. Its format and work–rest intervals can be customized to meet demands faced by public officers. Emergency professionals and first responders have reported insufficient time for additional training on proper and safe work practices [10]. Time-efficient HIFT sessions may help overcome this limitation and may be motivating due to enjoyment, challenge, or affiliation [18].

A key aspect of this proof of concept was to highlight the potential value of the PUSH protocol for training and health in public officers. Fernández-Fernández et al. [19] concluded that the physiological demands of different WODs met ACSM standards for vigorous-intensity exercise [20]. Exercise intensity plays a key role in driving adaptations, and recent research indicates that vigorous exercise yields greater benefits than moderate exercise [20]. Moreover, ACSM’s recommendation of 30 min of vigorous activity, which can be accumulated in 10 min bouts, aligned with the time frame of our WOD. In this study, PUSH was performed for approximately 10 min, placing our WOD at an intermediate duration compared with popular WODs.

Within the CrossFit^®^ model, benchmark workouts have different names; some of the most well-known include Karen, Fran, Grace, Cindy, Helen, and Murph. All of these WODs have been studied [15,19,21,22,23,24,25]. On the time–intensity spectrum, PUSH may be comparable to the Karen and Fran benchmark workouts in their “as prescribed” (RX) versions.

Sousa Neto et al. [22] reported that the Karen benchmark workout is completed in approximately the same time as PUSH (including CPR). In the studies by Leitão et al. [23] and Zarzosa et al. [15], which analyzed the physiological response to the Fran benchmark workout, athletes finished in a time similar to the PUSH METCOM. Blood lactate values fell within comparable interquartile ranges (IQR), age-adjusted mean and maximal HR were similar, and RPE was similar, characterizing the effort as “hard” to “very hard.” Thus, PUSH may be placed within the physical-demand spectrum of a popular benchmark workout.

CPR is a life-saving maneuver and, together with activation of emergency services, the most important intervention. Evidence has shown relationships between strength [26] and anaerobic endurance [27] and CPR quality. Thus, good physical condition may be relevant during prolonged resuscitations. Training under these conditions should be implemented in first responder protocols for fire and police personnel. An innovative aspect of this workout was the incorporation of CC on a medicine ball (commonly used in wall ball exercises). Although this component could be performed on commercial manikins, their cost and lack of specificity in this sporting context may limit dissemination. In contrast, using standard functional training equipment with resistance and texture similar to commercial CPR manikins can facilitate implementation both in training centers and in fire and police gyms.

### 4.1. Implications for Practice

In 2025, the ERCGL2025 executive summary introduced the “formula for survival,” defined as the interaction between science, education, and implementation to achieve optimal outcomes, and encouraged outreach and training at large-scale sporting events [28]. HIFT, in general, and CrossFit^®^ in particular, has millions of followers in more than 142 countries [12] and is an emerging sport that can contribute to the promotion and dissemination of CPR in sporting environments, such as firefighter and police competitions or the CrossFit^®^ Games. PUSH could join other established WODs with various performance standards (scaled, advanced, or RX), opening new avenues for research. This training model could be extended to other types of first responders or CrossFit^®^ athletes for the purpose of disseminating and promoting first aid and CPR.

Focusing on the findings of this proof of concept, our study showed that a high-intensity workout such as PUSH induced significant metabolic and cardiovascular stress while including elements common to rescue and CPR. This is relevant for designing evidence-based conditioning programs aimed at improving operational performance and safety among first responders.

### 4.2. Study Limitations

This study has several limitations that must be acknowledged. As a proof of concept, the sample included a small number of participants. Furthermore, it was tested only in public officers who practice CrossFit^®^, which may limit the generalizability of the results to the broader first responder population. The study also focused on a single type of High-Intensity Functional Training. Another point to note is that all participants were men, reflecting voluntary adherence in a context with low population density linked to the study focus (fire/police).

The workout was intentionally performed without a warm-up to simulate emergency conditions and adapt it to a real-world context. Although ecologically justified, this decision may influence heart rate responses, movement efficiency, injury risk, and the interpretation of physiological load.

From a gender perspective, both the methodology and analysis should include women, and researchers must commit to advancing more inclusive science. The CC were performed on a medicine ball (wall ball); this surface differs from a human torso and may involve a biomechanical position different from the standard for performing CPR.

Finally, although environmental conditions were controlled during testing, other unmeasured external factors may have influenced participant performance.

## 5. Conclusions

A realistic high-intensity WOD, such as the PUSH, which incorporates elements representative of public safety tasks performed by first responders during out-of-hospital emergencies, imposes a substantial metabolic demand in terms of blood lactate concentration, perceived fatigue, and peaks in maximum heart rate, making it a workout consistent with the intensity demands of rescue, assistance, and resuscitation interventions.

Experienced public safety personnel with a solid foundation in physical conditioning can maintain chest compressions using feedback, even under conditions of fatigue and submaximal lactate concentrations.

## Figures and Tables

**Table 2:** CPR variables.

Variables		Public Safety (*n* = 15) Median (IQR)	Police (*n* = 8) Median (IQR)	Fire (*n* = 7) Median (IQR)	*p*-Value (ES)
CC with adequate depth					
	BUY-IN	99 (98–100)	98 (96–99)	99 (99–100)	0.09 (0.45) ^ỻ^
	CASH-OUT	98 (98–99)	98 (97–99)	99 (98–99)	0.054 (0.50) ^ỻ^
Depth comparison		0.66 (0.11) ^χ^	0.68 (0.15) ^χ^	0.08 (0.65) ^χ^	
CC with adequate release					
	BUY-IN	89 (86–94)	89 (86–90)	96 (87–97)	0.16 (0.36) ^ỻ^
	CASH-OUT	91 (89–95)	90 (87–92)	96 (90–98)	0.13 (0.39) ^ỻ^
Release comparison		0.034 (0.61) †	0.12 (0.72) †	0.29 (0.43) †	
CC with adequate rate					
	BUY-IN	91 (88–95)	89 (79–92)	95 (91–97)	0.020 (1.36) *
	CASH-OUT	91 (86–96)	86 (80–91)	96 (94–98)	0.002 (2.06) *
Rate comparison		0.72 (0.09) †	0.81 (0.09) †	0.11 (0.72) †	
Compressions-only CPR quality					
	BUY-IN	93 (90–95)	90 (88–93)	96 (94–97)	0.011 (0.66) ^ỻ^
	CASH-OUT	94 (90–96)	91 (88–93)	96 (95–97)	0.016 (1.44) *
Score comparison		0.10 (0.43) ^χ^	0.36 (0.32) ^χ^	0.11 (0.70) †	

CC: Chest compressions. Buy-in: CC in baseline situation, at the start of the workout. Cash-out: CC under fatigue, at the end of the workout. IQR: Interquartile Range. ES: Effect size. * Independent samples *t* test (in brackets, effect size calculated with Cohen’s d), ^ỻ^ Mann–Whitney U (in brackets, effect size calculated with Rosenthal’s r), † paired samples *t* test (in brackets, effect size calculated with Paired Cohen’s d), ^χ^ Wilcoxon signed-rank test for paired samples (in brackets, effect size calculated with Rosenthal’s r). Significance level of *p* < 0.05 for all analysis.

## Data Availability

The original contributions presented in this study are included in the article. Further inquiries can be directed to the corresponding author.

## Supplementary Materials

The following supporting information can be downloaded at: https://www.mdpi.com/article/10.3390/jfmk11010060/s1.
