# Supplementary figures and images for: Public Safety Heroes (PUSH) Workout: Task-Specific High-Intensity Functional Training for Emergency Readiness in Fire and Police—Proof of Concept
Source: J Funct Morphol Kinesiol. 2026 Jan 30;11(1):60. doi: 10.3390/jfmk11010060 (PMC12921777; doi:10.3390/jfmk11010060)

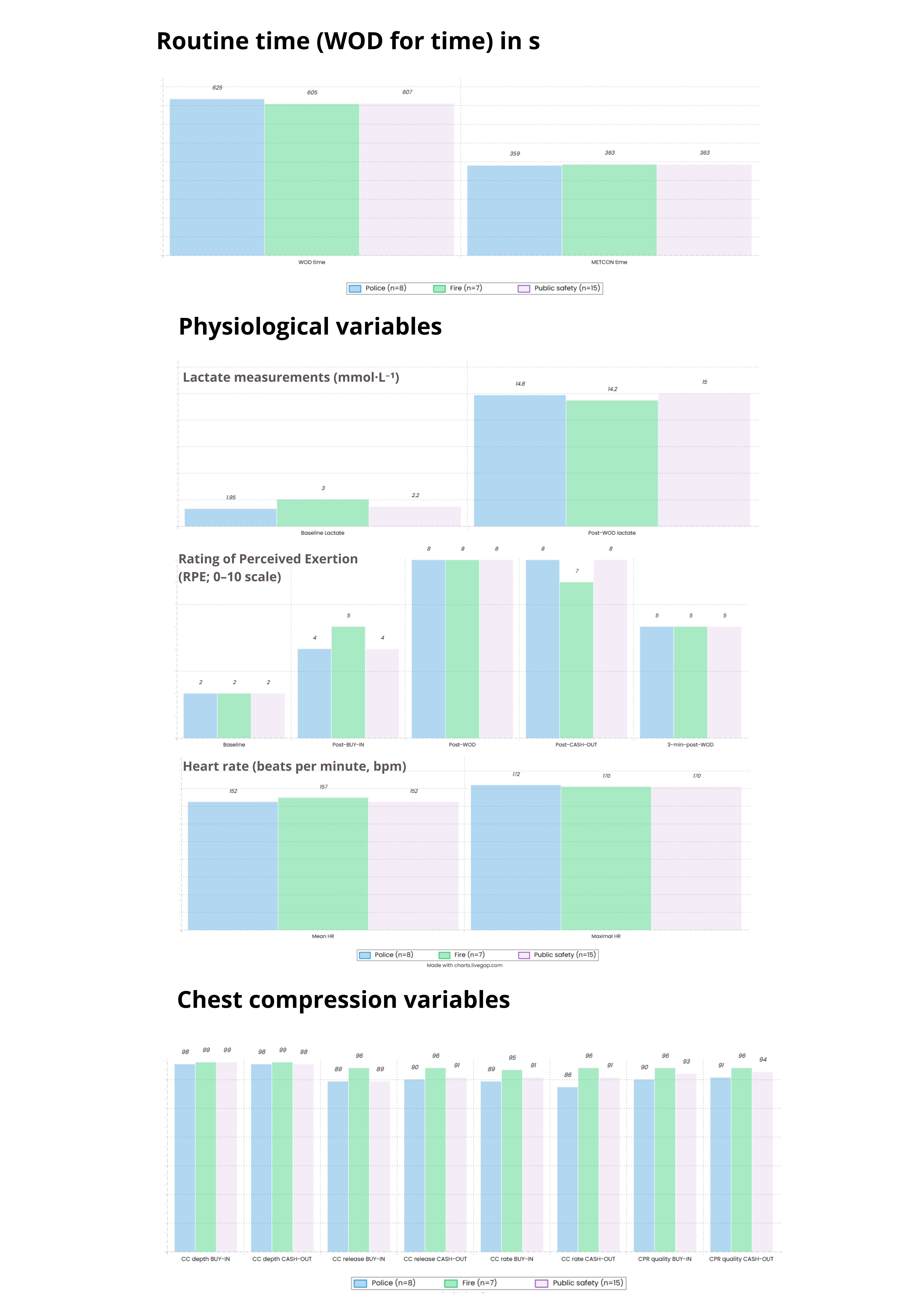

Supplement: Supplementary file 1 [file jfmk-11-00060-s001.zip › jfmk-4099118-supplementary-Figure S1.jpg]
